# S171. ALTERED WHITE MATTER CONNECTIVITY IN PATIENTS WITH SCHIZOPHRENIA USING PUBLIC NEUROIMAGING DATA FROM SCHIZCONNECT

**DOI:** 10.1093/schbul/sby018.958

**Published:** 2018-04-01

**Authors:** Sung Woo Joo, Woon Yoon, Seung-Hyun Shon, Saetbyeol Chat, JungSun Lee

**Affiliations:** 1 University of Ulsan College of Medicine, Asan Medical Center; 2 Asan Medical Center; 3 University of Ulsan College of Medicine

## Abstract

**Background:**

Several studies have produced a large body of evidence for white matter abnormalities related to schizophrenia. The literature has yet to achieve a state of consistency and reproducibility, and reported low integrity of white matter tracts vary between studies. Whole brain image study with large sample size is needed to address this issue. We investigated white matter integrity in connections between regions of interests (ROI) in the same hemisphere in patients with schizophrenia and healthy controls with public neuroimaging data from SchizConnect (http://schizconnect.org).

**Methods:**

A final data set was consisted of 129 healthy controls and 122 schizophrenia patients. For each diffusion weighted image (DWI), a two-tensor full-brain tractography was performed, and DWI images were parcellated by processing and registering the T1 images with FreeSurfer and the Advanced Normalization Tools. We extracted a total of 36 tracts in the both hemisphere connecting ROIs in the same hemisphere with white matter query language. We compared means of diffusion measures between patients and controls, and evaluated correlations with Letter-number sequencing (LNS) test, Vocabulary test, letter fluency test, category fluency test, and trails A of the Trail Making Test (TMT). The Benjamini-Hochberg procedure with false discovery rate (FDR) of 0.05 was used to correct for multiple comparisons.

**Results:**

We found a significant RD and TR increase of the left thalamo-occipital tracts and the right uncinate fascicle (UF), and a significant RD increase of the right middle longitudinal fascicle (MDLF), and the right superior longitudinal fascicle (SLF) ii in schizophrenia. There were correlations between the TR in the left thalamo-occipital tracts and letter fluency test, and the RD in the right SLF ii and LNS test, which did not survive after correction for multiple comparisons.

**Discussion:**

These results indicate widespread abnormalities of white matter fiber tracts in schizophrenia, contributing to the pathophysiology of schizophrenia.

